# Agreement among instruments of quantitative evaluation of the hard palate in children

**DOI:** 10.1590/2317-1782/20212020318

**Published:** 2021-10-25

**Authors:** Luana Cristina Berwig, Mariana Marquezan, Jovana de Moura Milanesi, Jessica Klöckner Knorst, Márlon Munhoz Montenegro, Amanda Cunha Regal de Castro, Eduardo Franzotti Sant’Anna, Thiago Machado Ardenghi, Ana Maria Toniolo da Silva

**Affiliations:** 1 Serviço de Fonoaudiologia, Hospital de Clínicas de Porto Alegre – HCPA – Porto Alegre (RS), Brasil.; 2 Departamento de Estomatologia, Faculdade de Odontologia, Universidade Federal de Santa Maria – UFSM – Santa Maria (RS), Brasil.; 3 Departamento de Fonoaudiologia, Curso de Fonoaudiologia, Universidade Federal de Santa Maria – UFSM – Santa Maria (RS), Brasil.; 4 Centro de Especialidades Odontológicas – CEO – Gravataí (RS), Brasil.; 5 Departamento de Ortodontia, Universidade Federal do Rio de Janeiro – UFRJ – Rio de Janeiro (RJ), Brasil.

**Keywords:** Hard Palate, Quantitative Assessment, Analytical Diagnostic, and Therapeutic Techniques and Equipment, Dimensional Measurement Accuracy, Child, Palato Duro, Avaliação Quantitativa, Diagnóstico analítico, técnicas e equipamentos terapêuticos, Precisão da Medição Dimensional, Criança

## Abstract

**Purpose:**

To evaluate the agreement among instruments of the quantitative evaluation of hard palate.

**Methods:**

This cross-sectional study was performed with a sample of 30 children aged 6 to 11 from Santa Maria, Southern Brazil. The instruments for palate measurements evaluated were: digital caliper, used directly in the oral cavity and in plaster casts, Korkhaus tridimensional bow, used directly in the oral cavity and in plaster casts, and Dolphin Imaging Software used for measurements in cone-beam computed tomography (CBCT). The agreement among different instruments was evaluated using the Intraclass Correlation Coefficient (ICC).

**Results:**

The means of all transversal dimensions obtained by cone-beam computed tomography were lower than those of the other instruments - the agreement values in the width between the canines and in the width between the first molars were lower when comparing the cone-beam computed tomography and the other instruments. In the width between the first and second premolars, all comparisons showed acceptable agreement values. Good concordance values were obtained when comparing the palate depth at the second premolar region when using a bow divider inside the oral cavity and in the cast.

**Conclusion:**

Most instruments presented satisfactory agreement in the measurements related to the transverse plane of the hard palate. However, when the vertical plane was evaluated, only the bow divider applied to both cast and oral cavity presented ideal agreement.

## RESUMO

**Objetivo:**

Avaliar a concordância entre instrumentos de avaliação quantitativa do palato duro.

**Método:**

Este estudo transversal foi realizado com uma amostra de 30 crianças de 6 a 11 anos de Santa Maria, sul do Brasil. Os instrumentos de medidas do palato avaliados foram: paquímetro digital, utilizado diretamente na cavidade oral e em modelos de gesso, arco tridimensional Korkhaus, usado diretamente na cavidade oral e em modelos de gesso, e Dolphin Imaging Software utilizado para medições em tomografia computadorizada de feixe cônico (CBCT). A concordância entre os diferentes instrumentos foi avaliada por meio do Coeficiente de Correlação Intraclasse (ICC).

**Resultados:**

As médias de todas as dimensões transversais obtidas pela tomografia computadorizada de feixe cônico foram menores do que as dos outros instrumentos - os valores de concordância na largura entre os caninos e na largura entre os primeiros molares foram baixos na comparação entre a tomografia computadorizada de feixe cônico e os demais instrumentos. Na largura entre o primeiro e o segundo pré-molar, todas as comparações apresentaram valores de concordância aceitáveis. Valores de concordância aceitáveis também foram obtidos ao comparar a profundidade do palato na região do segundo pré-molar com o uso de um divisor de arco dentro da cavidade oral e no gesso.

**Conclusão:**

A maioria dos instrumentos apresentou concordância satisfatória nas medidas relacionadas ao plano transverso do palato duro. Porém, quando avaliado o plano vertical, apenas o divisor de arco aplicado tanto no gesso quanto na cavidade oral apresentou concordância ideal.

## INTRODUCTION

The hard palate is the bone structure that divides the oral and nasal cavities. Its anatomy is closely related to orofacial functional activities. Therefore, its morphological analysis is characterized as an important part of the clinical evaluation of speech therapists working in Orofacial Motricity, and of dentists who work in Orthodontics. When their dimensions are altered, it can be predicted that oral functions and/or breathing will be impaired, to a greater or lesser degree^(1)^.

Subjective criteria are still the most used methods for morphological assessment of the hard palate in clinical practice. However, there are studies using different quantitative methods for the measurement of the hard palate. The caliper^(2,3)^ and the Korkhaus tridimensional bow divider^(4-6)^ are among the most frequently used instruments found in the literature. They may be applied directly inside the oral cavity^(4,6)^ or in plaster models^(2,3,5)^. The Caliper is a commonly used measuring instrument because it provides objective data in the evaluation of the hard palate and is advantageous because it is a simple, non-invasive, painless, and risk-free technique^(7,8)^. The three-dimensional Korkhaus bow divider is useful because it allows a three-dimensional assessment of the structures, however, it requires training for its use and is more complex to manage.

In addition, it is also possible to use softwares for the measurement of digital palate models^(9-11)^, or cone-beam computed tomography (CBTC) images^(11)^. Digital plaster models do not require physical storage and are also advantageous because they allow instant accessibility of information, as well as the ability to perform diagnostic configurations in a simple and electronically accurate manner^(10)^. Relation to CBTC, it contains low doses of radiation, a quick scanning time, and a high accuracy in the images obtained, also allowing the diagnosis through digital measurements^(11,12)^. Furthermore, there are even instruments designed specifically for the measurement of hard palate dimensions in plaster casts found in the literature^(13)^.

Knowledge about anthropometric measures contributes to the establishment of therapeutic conduct, as well as to the subsequent follow-up. Thus, given the different methods for hard palate measurement found in the literature, it is essential to investigate whether these instruments provide comparable results. Thus, the aim of this study is to verify the agreement among the instruments of quantitative evaluation of the vertical and transverse dimensions of the hard palate. The conceptual hypothesis was that different instruments yield similar and compatible results since all of them provide dimensions in millimeters.

## METHODS

### Ethical issues

The present study was registered and approved by the Ethics Committee in research on human beings of the Center of Health Sciences of the Federal University of Santa Maria under number 220.0.243.000-8. Only subjects who agreed to their participation, and whose parents or legal guardians signed Free and Informed Consent Forms were included.

### Study design and sample

This study is nested within a larger epidemiological survey carried out in 2015 in the city of Santa Maria, Southern Brazil. For the umbrella study, children were selected through a two-stage cluster process. The first stage consisted of schools (n = 9), followed by the inclusion of children enrolled in them. Schools were considered according to the sample weight and were distributed in the 8 administrative regions of the city. Subjects for this cross-sectional study were 30 children aged 6 to 11 years, with the full permanent upper-molar eruption and normal occlusion or Class I malocclusion. Subjects who presented evident cognitive syndromes or limitations, craniofacial malformation, or who had undergone orthodontic treatment were excluded.

Sample size calculation was made based on a pilot study. Pearson correlation test was used between hard palate measures obtained by different instruments. Based on the smaller “r” value (0.197) and a total number of 16 subjects, it was verified that 25 would be the minimum number of subjects to be included in the study.

### Calibration process

Assessments of the hard palate measurements were performed by two gold standard examiners in the area. One of the examiners (L.C.B.) performed all Caliper measurements within the oral cavity and in models and Korkhaus three-dimensional bow - within the oral cavity in models; and the other examiner (A.C.R.C.) performed all measurements through the CBCT. For the calibration process, hard palate measures were repeated after 7 days in 20% of the sample randomly selected, in order to assess intra-examiner reproducibility by means of the Intraclass Correlation Coefficient (ICC). The general ICC values ranged from 0.23-0.99. The specific values of ICC for each measure of the calibration process are described in Table 1.

**Table 1:** Assessment of intra-examiner reproducibility by means of the Intraclass Correlation Coefficient (ICC).

	**Transverse**	**Vertical**
**Caliper – within the oral cavity**	ICC ranged from 0.89 (first molar width) to 0.96 (first premolar width)	*Not possible to measure*
**Caliper – in models**	ICC ranged from 0.93 (second premolar width) to 0.99 (first premolar and first molar width)	ICC ranged from 0.67 (canine depth) to 0.93 (second premolar depth)
**Korkhaus tridimensional bow- – within the oral cavity**	ICC was 0.93 for canine width and 0.96 for second premolars width	ICC was 0.40 for canine depth and 0.87 for second premolar depth
**Korkhaus tridimensional bow- – in models**	ICC ranged from 0.97 (second premolar width) to 0.99 (first premolar width)	ICC ranged from 0.23 (canine depth) to 0.78 (second premolar depth)
**CBCT**	ICC ranged from 0.91 (first premolar width) to 0.92 (canine width)	ICC ranged from 0.71 (canine depth) to 0.96 (first and second premolar depth)

**Caption:** CBCT, cone-beam computed tomography

### Hard palate measurements

**Figure 1:**
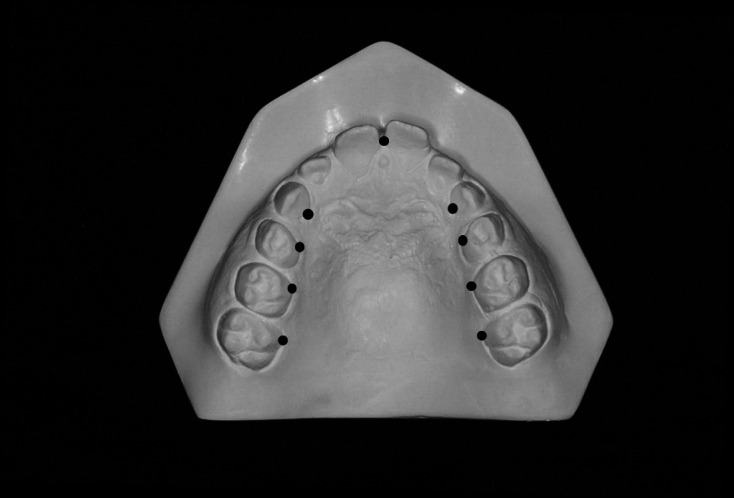
Reference points for hard palate measurements.

Hard palate measurements were obtained using the apical gingival margins^(3)^ of canine, premolar (or deciduous molar) and permanent molar teeth as reference (Figure 1). The width measurements were the obtained by the transverse dimensions’ assessment, in millimeters, between the apical gingival margins of each upper tooth. The depth measurements were obtained by the vertical dimension assessment, in millimeters, obtained from the median palatine raphe to the region uniting the points of the upper teeth. Measures were not taken when either one or both of the reference teeth were missing.

**Figure 2:**
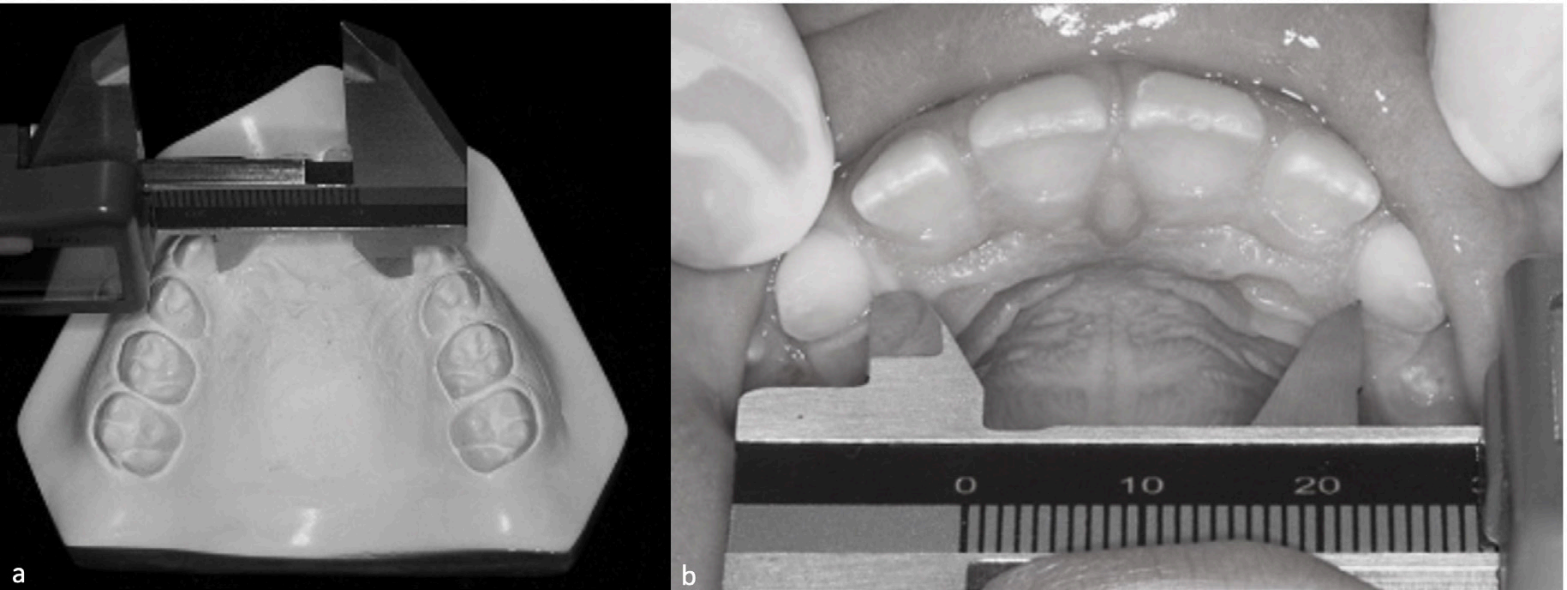
Hard palate transverse measurements with caliper in canines’ region: (a) plaster cast; (b) oral cavity.

**Figure 3:**
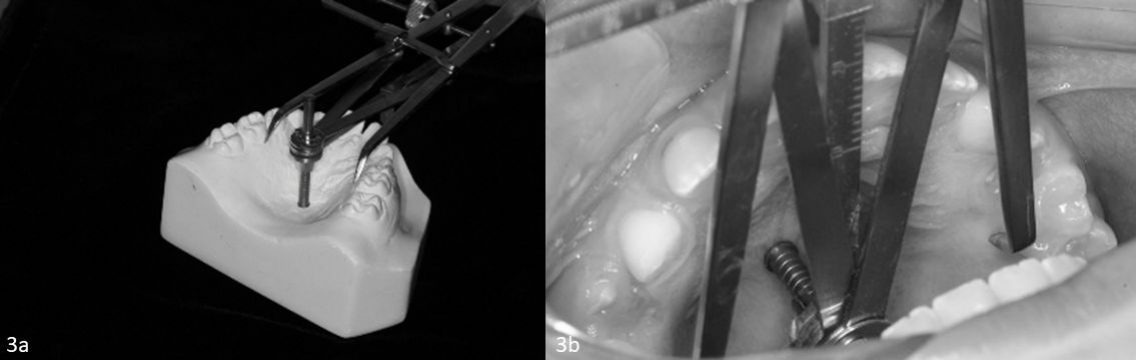
Hard palate measurements (transverse and vertical) with Korkhaus tridimensional bow divider in second molar region: (a) plaster cast; (b) oral cavity.

**Figure 4:**
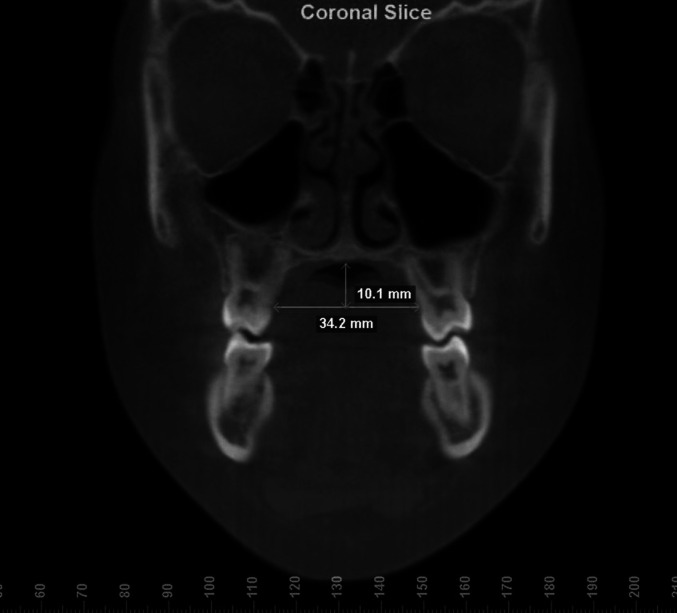
Width and depth hard palate measurements at first molar level with cone-beam computed tomography scan, coronal plane.

The instruments for palate measurements evaluated were: digital caliper (Digimess^®^, São Paulo, Brazil), used directly in the oral cavity and in plaster casts (Figure 2), Korkhaus tridimensional bow (Dentaurum^®^ Ispringen, Germany), used directly in the oral cavity and in plaster casts (Figure 3), and Dolphin Imaging Software (version 11, Chatsworth, USA) used for measurements in CBCT (Figure 4).

A trained examiner performed measurements in the oral cavity, with patient seated in dental equipment, and reclining chair at an angle of 45º, and under dental reflector lighting. In the same appointment, a dentist made plaster models of the upper-jaw dental arch of the subjects.

**Figure 5:**
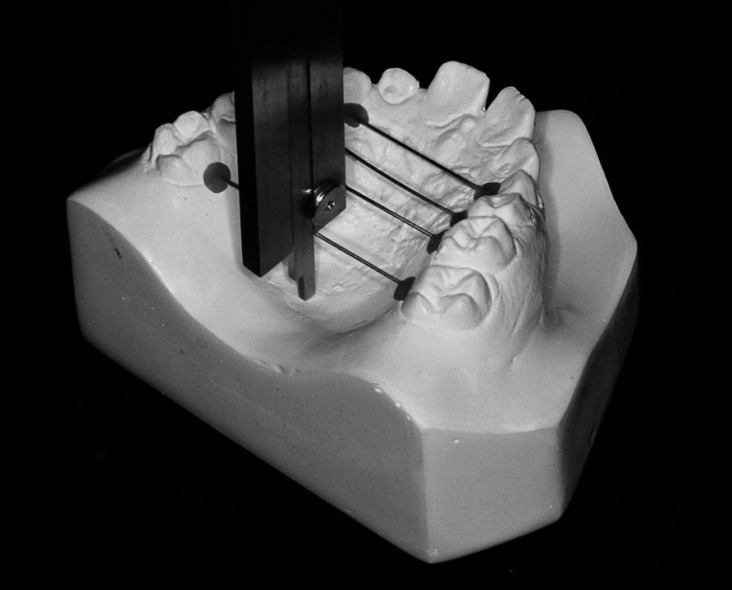
Hard palate depth measurement with caliper in plaster cast.

Using the casts, a trained examiner measured transverse and vertical dimension of the hard palates. It is important to emphasize that the caliper’s vertical measures (depth) could only be obtained in the plaster models because of the method adopted. A stainless steel orthodontic wire was cut so that its length would be equal to the transverse measure (width), and fixed with utility wax between the reference points at the level of each of the reference teeth (canine, premolar or deciduous molar and permanent molar). The depth was measured with the caliper rod, corresponding to the perpendicular measure from the median palatine line to the orthodontic wire uniting the region of each of the relevant teeth^(3)^ (Figure 5). The diameter of the stainless steel (0.5mm) was subtracted from the vertical measures.

Within a week after the plaster models were made and the measures taken within the oral cavity, each subject underwent a CBCT scan (i-CAT Cone Beam 3-D Imaging System, PA, USA), which allowed cranium and facial scanning for future computerized reconstruction. The tridimensional images (DICOM) captured were exported to Dolphin Imaging Software and a previously trained examiner performed the measurements in coronal plane (Figure 4).

### Data analysis

Data were analyzed by software SPSS 20.0 (Statistical Package for the Social Sciences). The agreement study among different instruments for the different measurements of the hard palate was undertaken by calculation of the ICC. Reliable values must be superior to 0.70^(14)^.

## RESULTS

The sample consisted of 30 children evaluated for measurements of the hard palate. Tables 2 and 3 display the mean and standard deviation, the difference between means, and the agreement between the transverse/width measures (Table 2) and the vertical measures (Table 3) obtained by different assessment methods.

**Table 2:** Mean and standard deviation, difference between means and agreement among width measures obtained by different methods.

**Measure**	**Method**	**Mean ± SD**	**Comparison between methods**	**Means difference (mm)**	**Agreement ICC (CI 95%)**
**Canine Width**	Bow divider OC	28.32 ± 2.21	bow divider OC *vs.* caliper OC	0.83	0.85 (0.46-0.94)*
			bow divider OC *vs.* CT scan	2.00	0.59 (-0.08-0.86)*
	Caliper OC	27.49 ± 2.40	bow divider OC *vs.* bow divider PM	0.39	0.90 (0.79-0.96)*
			bow divider OC *vs.* caliper PM	0.85	0.88 (0.42-0.96)*
	CT scan	26.32 ± 2.12	caliper OC *vs.* CT scan	1.17	0.69 (0.37-0.87)*
			caliper OC *vs.* bow divider PM	0.44	0.91 (0.69-0.97)*
	Bow divider PM	27.93 ± 2.34	caliper OC *vs.* caliper PM	0.02	0.95 (0.89-0.97)*
			CT scan *vs.* bow divider PM	-1.61	0.67 (0.05-0.89)*
	Caliper PM	27.47 ± 2.24	CT scan *vs.* caliper PM	-1.15	0.75 (0.32-0.91)*
			bow divider PM *vs.* caliper PM	0.46	0.96 (0.59-0.99)*
**First Premolar Width**	Caliper OC	29.14 ± 2.39	caliper OC *vs.* CT scan	0.48	0.96 (0.88-0.98)*
			caliper OC *vs.* bow divider PM	-0.79	0.92 (0.22-0.98)*
	CT scan	28.66 ± 2.33	caliper OC *vs.* caliper PM	-0.32	0.96 (0.89-0.98)*
	Bow divider PM	29.93 ± 2.49	CT scan *vs.* bow divider PM	-1.27	0.88 (0.05-0.97)*
	Caliper PM	29.46 ± 2.39	CT scan *vs.* caliper PM	-0.80	0.95 (0.62-0.98)*
			bow divider PM *vs.* caliper PM	0.47	0.96 (0.63-0.99)*
**Second Premolar Width**	Bow divider OC	33.68 ± 2.69	bow divider OC *vs.* caliper OC	0.77	0.92 (0.46-0.97)*
			bow divider OC *vs.* CT scan	1.39	0.85 (0.13-0.96)*
	Caliper OC	32.91 ± 2.44	bow divider OC *vs.* bow divider PM	0.34	0.94 (0.84-0.97)*
			bow divider OC *vs.* caliper PM	0.84	0.90 (0.15-0.97)*
	CT scan	32.29 ± 2.47	caliper OC *vs.* CT scan	0.62	0.91 (0.76-0.96)*
			caliper OC *vs.* bow divider PM	-0.43	0.95 (0.88-0.98)*
	Bow divider PM	33.34 ± 2.36	caliper OC *vs.* caliper PM	0.07	0.96 (0.93-0.98)*
			CT scan *vs.* bow divider PM	-1.05	0.89 (0.48-0.96)*
	Caliper PM	32.84 ± 2.41	CT scan *vs.* caliper PM	-0.55	0.95 (0.87-0.98)*
			bow divider PM *vs* caliper PM	0.50	0.96 (0.45-0.99)*
**First Molar Width**	Caliper OC	36.62 ± 2.66	caliper OC *vs.* CT scan	2.72	0.63 (-0.08-0.89)*
			caliper OC *vs.* bow divider PM	-2.71	0.93 (0.84-0.97)*
	CT scan	33.90 ± 2.56	caliper OC *vs.* caliper PM	0.96	0.86 (0.17-0.96)*
	Bow divider PM	36.33 ± 2.47	CT scan *vs.* bow divider PM	-2.43	0.72 (-0.07-0.92)*
	Caliper PM	35.66 ± 2.49	CT scan *vs.* caliper PM	-1.76	0.84 (0.04-0.95)*
			bow divider PM *vs.* caliper PM	0.67	0.95 (0.07-0.99)*

* p<0.01

**Caption:** OC=measures taken within the oral cavity; PM=measures taken in plaster models; CT= computed tomography; SD, standard deviation; ICC, intraclass correlation coefficient; CI, confidence interval; *vs.=versus*

**Table 3:** Agreement among hard palate measurement instruments for vertical measures measured within the oral cavity, CT scan and plaster model.

**Measure**	**Method**	**Mean ± SD**	**Comparison between methods**	**Means difference (mm)**	**Agreement** **ICC (CI 95%)**
**Canine Depth**	Bow divider OC	1.87 ± 0.75	bow divider OC *vs.* CT scan	-2.03	0.09 (-0.10-0.39)
			bow divider OC *vs.* bow divider PM	0.19	0.03 (-0.36-0.42)
	CT scan	3.90 ± 1.45	bow divider OC *vs.* caliper PM	-1.86	0.10 (-0.07-0.37)*
	Bow divider PM	1.68 ± 0.72	CT scan *vs.* bow divider PM	2.22	0.01 (-0.07-0.21)
	Caliper PM	3.73 ± 1.09	CT scan *vs.* caliper PM	0.17	0.41 (-0.06-0.72)*
			bow divider PM *vs.* caliper PM	-2.05	0.07 (-0.05-0.29)*
**First Premolar Depth**	CT scan	9.69 ± 1.81	CT scan *vs.* bow divider PM	3.36	0.23 (-0.06-0.62)**
	Bow divider PM	6.33 ± 1.55	CT scan *vs.* caliper PM	1.25	0.56 (0.12-0.80)**
	Caliper PM	8.44 ± 1.55	bow divider PM *vs.* caliper PM	-2.11	0.43 (-0.06-0.79)**
**Second Premolar Depth**	Bow divider OC	9.93 ± 1.58	bow divider OC *vs.* CT scan	-1.07	0.61 (-0.03-0.86)**
			bow divider OC *vs.* bow divider PM	-0.10	0.74 (0.51-0.87)**
	CT scan	11.00 ± 1.66	bow divider OC *vs.* caliper PM	-1.76	0.49 (-0.07-0.82)**
	Bow divider PM	10.03 ± 1.92	CT scan *vs.* bow divider PM	0.97	0.52 (0.12-0.78)**
	Caliper PM	11.69 ± 1.75	CT scan *vs.* caliper PM	-0.69	0.69 (0.16-0.88)**
			bow divider PM *vs.* caliper PM	-1.66	0.58 (-0.04-0.83)**
**First Molar Depth**	CT scan	9.40 ± 1.58	CT scan *vs.* bow divider PM	-0.65	0.66 (0.33-0.84)**
	Bow divider PM	10.05 ±1.89	CT scan *vs.* caliper PM	-2.29	0.38 (-0.08-0.75)**
	Caliper PM	11.69 ± 1.75	bow divider PM *vs.* caliper PM	-1.64	0.59 (-0.09-0.85)**

* p<0.05;

** p<0.01

**Caption:** SD, standard deviation; ICC, intraclass correlation coefficient; CI, confidence interval; *vs.=versus;* OC=measures taken within the oral cavity; PM=measures taken in plaster models; CT= computed tomography

Regarding transverse measures (Table 2), smaller agreement values in canine width were observed when CBCT scan was compared to bow divider within the oral cavity (ICC=0.59), bow divider in models (ICC=0.67) and caliper within the oral cavity (ICC=0.69). Similarly, smaller agreement values were found in width between first molars in the comparison between CBCT scan with caliper within the oral cavity (ICC=0.63) and bow divider in models (ICC=0.72). The means of all transverse dimensions obtained by CBCT scan were smaller than those of the other instruments. In the comparison of width between first and second premolars among the different methods tested, all agreement values were acceptable (ICC between 0.85 and 0.96).

Regarding vertical measures (Table 3), only one agreement value was above acceptance level (>0.70). The only acceptable agreement value obtained was depth at second premolar level, in the comparison between values taken by bow divider within the oral cavity and in model (ICC=0.74).

## DISCUSSION

This study was undertaken as a comparison between agreements for different methods that may be used in the measurement of the hard palate, seeking to assess whether different methods may provide comparable results, given that there is as yet no gold standard in the literature for this type of evaluation. Our findings partially confirm the conceptual hypothesis, since the measurements were only comparable for transverse measurements. Although some previous studies have undertaken the comparison of hard palate measurements among different variables, such as sex, age group, ethnicity, and oral habits^(4-6)^, the agreement among different methods has not been explored yet.

Regarding the intra-examiner reproducibility of the methods under evaluation in this study, the values obtained were within acceptable limits for all width dimensions. On the other hand, in the analysis of vertical measures, agreement values were progressively smaller toward the frontal region of the mouth. At canine level, the bow divider yielded values much below acceptable levels. With the use of the caliper for depth measurement in models, agreements values at canine and first premolar levels were higher than those obtained with the bow divider, but were still beneath acceptable levels. The low agreement values between test and retest depth measurements in the frontal hard palate region may be explained by the difficulty in finding the ideal support point for the rods of the instruments in a region with a more curvilinear anatomy^(15)^. The analysis of intra-examiner reproducibility may lead to the inference that frontal hard palate depth measurements with bow divider and caliper may not be reliable, and does not support the internal validity of the studies. On the other hand, when the objective is to study the vertical dimensions of the frontal hard palate, the use of CBCT scans seems to be more appropriate.

In the analysis of the results for transverse measurements, the means of the transverse dimensions obtained by CBCT scans were smaller than those obtained by bow divider and caliper both within the oral cavity and in models. One possible explanation for this would be that it is difficult to detect soft tissues by conventional CBCT scans. The literature presents one study proposing a way to eliminate the overlap of structures with a view to make the visualization of position and thickness of gingival tissue easier^(16)^, thus reinforcing the difficulty in identifying this tissue in conventional exams. When CBCT scans were compared to the other instruments, it was verified that canine width presented concordance levels below the expected in comparison with bow divider (both within the oral cavity and in models), and with caliper within the oral cavity. One possible explanation is that the low agreement levels found between CBCT scan and the other instruments is related to the identification of the reference points used in this study. The coronal plane is not influenced by soft tissues, whereas, in the clinical hard palate measurement with caliper and bow divider, the gingival margin must be taken as reference point, which may justify the low agreement among methods.

On the other hand, for width measurements between first and second premolars, all comparisons among methods presented acceptable agreement levels, including comparison with CBCT scan. It may be suggested that, at premolar or deciduous molar levels, the reference point used in CBCT scans is closer to the one clinically identified in the gingival margin. At premolar level, there is higher stability regarding the thickness of the epithelium covering the palate, as well as regarding the position of the gingival margin^(17)^.

Regarding the width between first molars, agreement values were below expectation in the comparison between CBCT scan and caliper within the oral cavity. This difference may be explained not only by the impossibility to locate the gingival margin in tomography planes, but also by the difficulty in locating the apical gingival margin at first molar level in the oral cavity, due to the more distal position of these teeth in relation to the others considered in this study. This hypothesis is corroborated by the higher agreement value found for the comparison between CT scan measurements with those taken by caliper in model.

Regarding vertical measures, most agreement values among instruments were below expectation. The only acceptable agreement value was depth obtained at second premolar level, when compared to the value obtained for the use of bow divider both within the oral cavity and in the model. It may be observed from this that the hard palate depth measurement instruments under consideration in this study may yield differing results. It is believed that the difficulty in obtaining compatible values among frontal hard palate depth measurement instruments is due to the variability of palatine roughness in the region of the incisive papilla^(18)^, and to the differences between in diameter the bow divider’s vertical rod and the caliper’s vertical rod area^(7,8)^.

From the comparative analysis of the different instruments under consideration in this study for hard palate width measurement, it was verified that most of the comparisons (87.5%) presented agreement values within acceptable levels, the highest being those of vertical measures at premolar levels. Among the instruments in question, the caliper may be the best choice for measurements both within the oral cavity and in plaster models, as it is a low-cost, precise and easy-to-handle tool^(7)^, which displays results digitally. On the other hand, it was verified that most comparisons among vertical measures for the various instruments presented low agreement values, thus disallowing this study’s hypothesis. Acceptable agreement values were observed only at second premolar levels in comparison between vertical measures obtained with bow divider both within the oral cavity and in the model.

This study has some limitations. The analysis undertaken in this study does not allow inference of which instrument would be most suitable for hard palate depth measurement. It is believed that some anatomic peculiarities, such as the inherent structural concavity, and different epithelium features and degrees of thickness may have contributed to the lack of equivalence among measures by different instruments. It is necessary to take into account the specificities of the instruments used to obtain the depth dimensions at the level of each of the teeth that were considered. Different means were found in the comparison among the different methods, and it was observed that depth values taken by bow divider tend to be undervalued in relation to those obtained by other instruments.

The use of the bow divider for depth measurement at canine level both within the oral cavity and in model and of the caliper for depth measurement at canine and first premolar levels yielded non-reproducible measures. This indicates that caution is necessary in relation to these instruments for the assessment of the frontal region of the hard palate depth. More research is necessary on the subject in order to improve palatine vertical measures, since the agreement values obtained in the present study were below the ideal.

## CONCLUSION

Our findings showed that most hard palate measurement instruments present satisfactory agreement for transverse values. However, for vertical dimensions, a low agreement was verified among most instruments.
